# *S*-Adenosyl-L-Homocysteine Hydrolase (SAHH): Structure, Function, and Applications

**DOI:** 10.3390/biom16071010

**Published:** 2026-07-10

**Authors:** Jinsha Huang, Qingpu Chen, Haihua He, Kai Du, Zhangli Hu

**Affiliations:** 1 College of Life Sciences and Oceanography, Shenzhen University, Shenzhen 518060, China; 2 Research & Development Department, Shenzhen New Industries Biomedical Engineering Co., Ltd. (Snibe), Shenzhen 518122, China

**Keywords:** *S*-adenosyl-L-homocysteine hydrolase, SAHH, molecular structure, cellular methylation, catalytic mechanism, protein–ligand interaction, drug development, clinical biomarkers, enzymatic synthesis

## Abstract

*S*-adenosyl-L-homocysteine hydrolase (SAHH) is an evolutionarily conserved enzyme present in eukaryotes, bacteria, and archaea. As the rate-limiting enzyme in the methionine cycle, it catalyzes the reversible hydrolysis of *S*-adenosyl-L-homocysteine (SAH) to adenosine and homocysteine, thereby modulating the *S*-adenosylmethionine/SAH ratio and cellular methylation potential. Dysregulation of SAHH activity is causally linked to cancer, cardiovascular disorders, and neurodegenerative conditions. This review systematically examines the biological distribution, catalytic mechanisms, structural architecture, and regulation of SAHH across diverse species. We highlight lineage-specific adaptations—including C-terminal truncation, a 40-residue substrate-binding-domain insertion, and a His-Phe molecular gate—that fine-tune substrate preference, cofactor affinity, and thermostability, with metal ions and NAD^+^ serving as key modulators of activity and conformational dynamics. These variations exemplify an evolutionary trade-off between catalytic efficiency and structural rigidity, particularly pronounced in archaeal and thermophilic orthologs. Collectively, these insights underpin the enzyme’s multifaceted translational value: SAHH serves as a therapeutic target for diverse diseases (e.g., cancer, viral infections, tuberculosis), a source of diagnostic/prognostic biomarkers (e.g., plasma homocysteine and SAH/SAM ratio), and a versatile biocatalyst for synthesizing pharmaceutical-grade adenosine and its derivatives. By integrating mechanistic, structural, and evolutionary perspectives, this review establishes a unified framework that explains these functional adaptations and their translational implications. This framework guides the rational development of SAHH-targeted inhibitors, diagnostic tools, and engineered biocatalysts, with broad applications in precision medicine and biotechnology.

## 1. Introduction

*S*-adenosyl-L-homocysteine hydrolase (SAHH, EC 3.3.1.1) is a highly conserved enzyme central to methylation metabolism, ubiquitously found in numerous eukaryotes and prokaryotes [1,2]. It reversibly hydrolyzes *S*-adenosyl-L-homocysteine (SAH) into adenosine and homocysteine (Hcy), thereby regulating the intracellular *S*-adenosylmethionine (SAM)-to-SAH ratio—a key determinant of cellular methylation [3]. Through this mechanism, SAHH influences epigenetic patterning and the methylation of diverse substrates, including small molecules (e.g., norepinephrine, catecholamines, and phospholipids) and macromolecules (e.g., proteins, nucleic acids, polysaccharides, and ribosomes) [4,5]. Dysregulation of SAHH is implicated in multiple diseases, such as vascular disease, myopathy, fatty liver, tumorigenesis, renal insufficiency, and diabetic nephropathy [6,7]. Given its physiological significance, SAHH is a promising therapeutic target for antiviral and antitumor drug development. It is a valuable tool in enzymatic synthesis for the production of adenosine and its derivatives in biotechnology and pharmaceutical research, and its reaction products also serve as potential biomarkers for disease diagnosis and prognosis [8,9].

In recent years, SAHH research has expanded rapidly, marked by important insights across structural biology, drug discovery, and disease biology. High-resolution structures of SAHH from extremophiles, including the first dimeric bacterial enzyme, have uncovered species-specific substrate-recognition fingerprints and C-terminal regulatory elements that distinguish archaeal, bacterial, and eukaryotic enzymes [1,10]. Concurrently, modern virtual screening campaigns have delivered novel, selective SAHH inhibitors against pathogens such as *Mycobacterium tuberculosis* and *Naegleria fowleri*, opening new avenues for antimicrobial development [11,12]. Beyond its classical role as a methylation gatekeeper, SAHH has been redefined as a direct participant in autophagy regulation, non-coding RNA-mediated glioblastoma growth, and post-translational modulation of metabolic enzymes [6,13,14,15]. Together, these discoveries reveal an important functional richness and establish SAHH as a multi-dimensional hub that integrates metabolism, epigenetics, and cellular signaling.

Despite this progress, current SAHH research remains fragmented across structural biology, enzymology, medicinal chemistry, and clinical diagnostics [16,17]. High-resolution structures and mechanistic studies have largely clarified the Rossmann-fold domains, the molecular gate that controls active-site accessibility, and the catalytic pathway; meanwhile, plasma Hcy and the SAH/SAM ratio serve as clinical risk indicators, and inhibitors such as 3-deazaadenosine analogs show therapeutic promise. However, a systematic framework integrating molecular features with practical applications is lacking, impeding synergistic progress in rational inhibitor design, multi-omics biomarker development, and industrial enzyme engineering. In this review, we systematically consolidate recent advances in SAHH across diverse species. We cover topics spanning substrate/cofactor recognition, species-specific insertions, catalytic mechanisms, metal ion regulation and cofactor activation. Our aim is to connect fundamental molecular insights with the demands of clinical and industrial applications and to provide a coherent resource for researchers in medicinal chemistry, precision medicine, and biomanufacturing.

By integrating mechanistic, structural, and evolutionary perspectives, this review provides a unified framework that connects molecular details to functional applications. This framework is intended to guide researchers in exploiting SAHH as a therapeutic target, a diagnostic biomarker, and a biocatalyst, ultimately accelerating the translation of fundamental discovery into clinical and industrial innovations. The review is organized as follows. Section 2 describes the catalytic mechanism and the phylogenetic divergence in substrate specificity. Section 3 outlines standard activity assays. Section 4 details the protein structure, while Section 5 well discusses the molecular gate, metal-ion modulation, and interactions with substrates and the NAD^+^ cofactor. Section 6 examines the biological distribution and evolutionary innovations of SAHH across species. Section 7 covers translational applications in drug development (Section 7.1), clinical biomarkers (Section 7.2), and enzymatic synthesis (Section 7.3). Section 8 concludes with future perspectives.

## 2. Catalytic Mechanism and Reaction

### 2.1. Catalytic Mechanism

**Figure 1:**
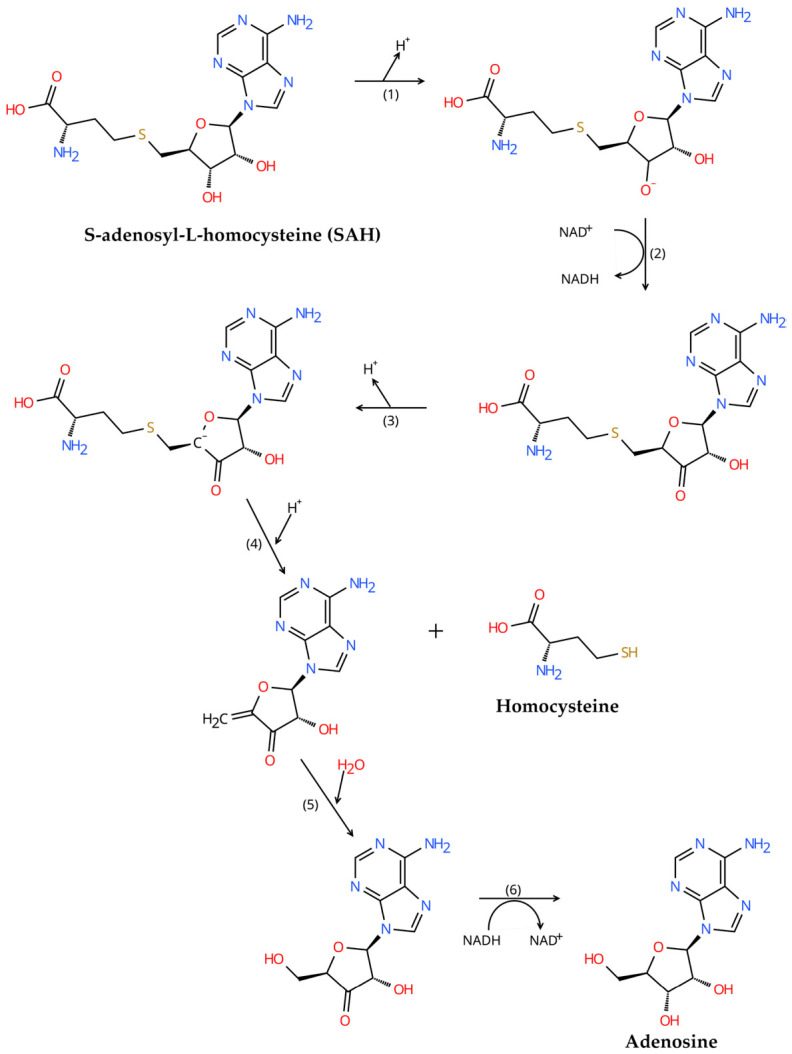
Catalytic mechanism of SAHH-mediated hydrolysis of SAH to homocysteine and adenosine [20]. Schematic generated with Ketcher (https://lifescience.opensource.epam.com/KetcherDemoSA/index.html, accessed on 25 June 2026) [21].

The catalytic mechanism for SAH hydrolysis was first elucidated by Palmer and Abeles in 1979 [18]. The process is coupled with the redox cycling of the noncovalently bound cofactor NAD^+^. Phylogenetic analyses indicate that both the catalytic mechanism and the key residues involved are exceptionally well-conserved across species [19]. Using SAHH from *Lupinus luteus* (*Ll*SAHH) as a representative model [20], the reaction proceeds through the following six steps, which are illustrated in Figure 1:

(1) Deprotonation: The ε-amino group of K235 acts as a general base, abstracting a proton from the 3′-OH of the adenosine moiety in SAH.
(2) Oxidation: The nucleophilicity of K235 is enhanced through hydrogen bonds with N240-Oδ1, N230-Oδ1, and the carbonyl oxygen of E205. This deprotonation facilitates the oxidation of the 3′-carbon by NAD^+^, yielding a 3′-keto intermediate (3′-keto-AdoHcy) and NADH.
(3) Enolate formation: The 3′-keto group increases the acidity of the C4′ proton, enabling its abstraction by the carboxylate of D139, generating a C4′-carbanion (enolate) intermediate.
(4) Elimination of Hcy: The imidazole ring of H62 donates a proton to the sulfur atom of Hcy, facilitating β-elimination and cleavage of the thioether linkage. This results in the release of Hcy and the formation of 3′-keto-4′,5′-didehydroadenosine.
(5) Hydration: A water molecule adds across the C4′–C5′ double bond via Michael addition, producing 3′-keto-adenosine.
(6) Reduction and product release: The 3′-keto group is reduced back to a hydroxyl by NADH, regenerating NAD^+^ and yielding adenosine. The release of adenosine induces the a conformational change in the enzyme from the closed to the open state [22].

### 2.2. Catalytic Reaction and Substrate Specificity

SAHH is canonically defined by its reversible hydrolysis of SAH to adenosine and Hcy, a reaction central to cellular methylation homeostasis [16,23]. Notably, emerging evidence reveals pronounced functional divergence among SAHH orthologs, particularly those derived from archaea and extremophilic bacteria, with the most striking variation observed in substrate specificity [1]. This functional split is tightly coupled to phylogeny and encoded by a conserved fingerprint motif (HxTxE(Q) or HxExK) in the substrate-binding domain [1].

**Figure 2:**
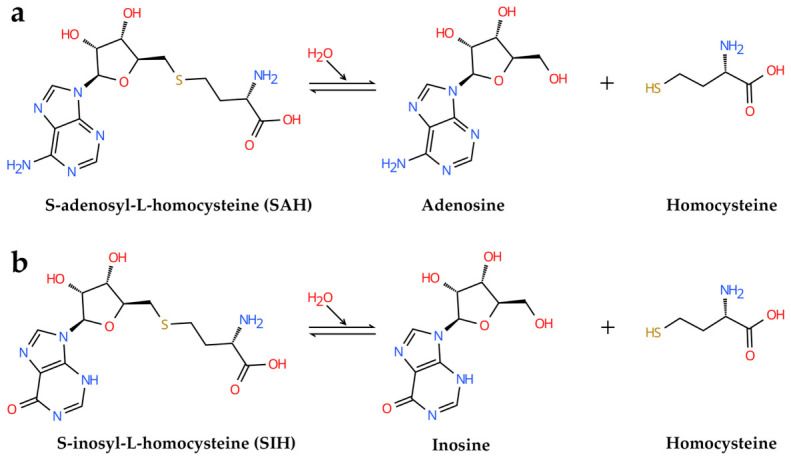
Catalytic reactions mediated by SAHH. Schematic generated with Ketcher (https://lifescience.opensource.epam.com/KetcherDemoSA/index.html, accessed on 25 June 2026) [21]. (**a**) Hydrolysis of SAH to adenosine and homocysteine; (**b**) hydrolysis of SIH to inosine and homocysteine.

Orthologs bearing the HxTxE(Q) motif, found in eukaryotes and most bacteria, retain the canonical SAH hydrolase activity [1]. They catalyze the reversible conversion of SAH to adenosine and Hcy (Figure 2a), driving the reaction toward hydrolysis in vivo to relieve SAH-mediated feedback inhibition of SAM-dependent methyltransferases, thereby sustaining the classical methyl cycle [16].

In contrast, orthologs with the HxExK motif are characteristic of *Thermotoga maritima* and euryarchaeal species such as *Methanocaldococcus jannaschii*, *Methanococci*, and *Thermococci* and exhibit a marked preference for hypoxanthine-containing substrates. They reversibly cleave *S*-inosyl-L-homocysteine (SIH) into inosine and Hcy (Figure 2b) [24]. This SIH preference supports an alternative SAM regeneration pathway wherein SAH is first deaminated to SIH prior to hydrolysis. As established by Koeppl et al., the mutually exclusive HxTxQ(E) and HxExK motifs directly encode substrate specificity, providing a structural basis for the phylogenetic divide between canonical and alternative methyl metabolism [1].

The difference in substrate specificity is not a minor biochemical variation but a deeply rooted evolutionary specialization. This functional bifurcation maps onto a deep phylogenetic split within Archaea: Crenarchaeota retain canonical SAH hydrolase activity, whereas Euryarchaeota have evolved SIH preference as an adaptation to alternative SAM salvage pathways involving inosine-based intermediates [1]. The structural basis and evolutionary drivers of this substrate rewiring are discussed in Section 6. Given the physiological centrality of SAH hydrolysis, this review focuses exclusively on its catalysis.

## 3. Standard Assays for Hydrolytic Activity Measurement

### 3.1. Spectrophotometric Assay Based on Hcy Detection (Ellman’s Method)

Hcy possesses a free sulfhydryl group that reacts with Ellman’s reagent (5,5′-dithiobis-(2-nitrobenzoic acid), DTNB) to yield 2-nitro-5-thiobenzoate (TNB^2−^), a yellow-colored product detectable at 412 nm [25]. This spectrophotometric method has been widely adopted for monitoring SAHH activity by quantifying Hcy production. A standard curve is prepared using Hcy standards (1–200 μM, prepared in 10 mM phosphate buffer, pH 7.4). Each 200 μL aliquot of standard is mixed with 100 μL of 4 mM DTNB and incubated for 30 min at 37 °C in the dark, followed by absorbance measurement at 412 nm.

For enzymatic activity assessment, a reaction mixture containing a defined amount of purified SAHH and 1 mM SAH is incubated at 37 °C for 1 h [26]. The reaction is terminated by ultrafiltration using a 10 kDa molecular weight cut-off centrifugal device to remove protein. An aliquot (200 μL) of the filtrate is reacted with 100 μL of 4 mM DTNB under conditions identical to those for the standards. The concentration of generated Hcy is determined by interpolation from the standard curve. The extinction coefficient of NTB^2−^ was determined to be 14,150 M^−1^·cm^−1^ under standard buffer conditions and 13,700 M^−1^·cm^−1^ in denaturing buffers containing 6 M guanidine hydrochloride or 8 M urea [27].

One unit (U) of SAHH activity is defined as the amount of enzyme required to produce 1 μmol of Hcy per minute under the specified conditions. In summary, Ellman’s method offers operational simplicity and rapid sample processing. However, a major drawback is its lack of specificity for Hcy over other thiol-containing compounds, which may compromise sensitivity and accuracy [26].

### 3.2. High-Performance Liquid Chromatography (HPLC) for SAH and Adenosine Quantification

HPLC enables precise quantification of SAHH activity by monitoring the consumption of substrate SAH or the production of adenosine [1,28]. The retention times of SAH and adenosine are 8.0 min and 7.9 min, respectively. Chromatographic separation is performed on an Agilent 1100 series HPLC system equipped with an ISAspher SCX 100-5 column (250 mm × 4.6 mm, 5 µm; ISERA GmbH, Düren, Germany). The mobile phase consists of 40 mM sodium acetate (pH 4.2, buffer A) and acetonitrile (buffer B). Elution is carried out with the following gradient program: 0–4 min, 100% A at 1.3 mL/min; 4–10 min, 70% A and 30% B at 1.1 mL/min; 10–20 min, 100% A at 1.3 mL/min for column re-equilibration. The injection volume is set to 10 µL.

The HPLC method provides high sensitivity and reproducibility, making it suitable for precise enzymatic characterization, especially for time-course analysis. However, it requires specialized instrumentation, advanced operational expertise, and careful optimization of the mobile phase and column conditions. Nevertheless, its quantitative and high-resolution measurements render it invaluable for detailed kinetic studies. Therefore, the choice between Ellman’s assay and HPLC should be guided by specific experimental requirements regarding accuracy, throughput, and resource availability.

## 4. Structural Features

### 4.1. Overall Architecture

**Figure 3:**
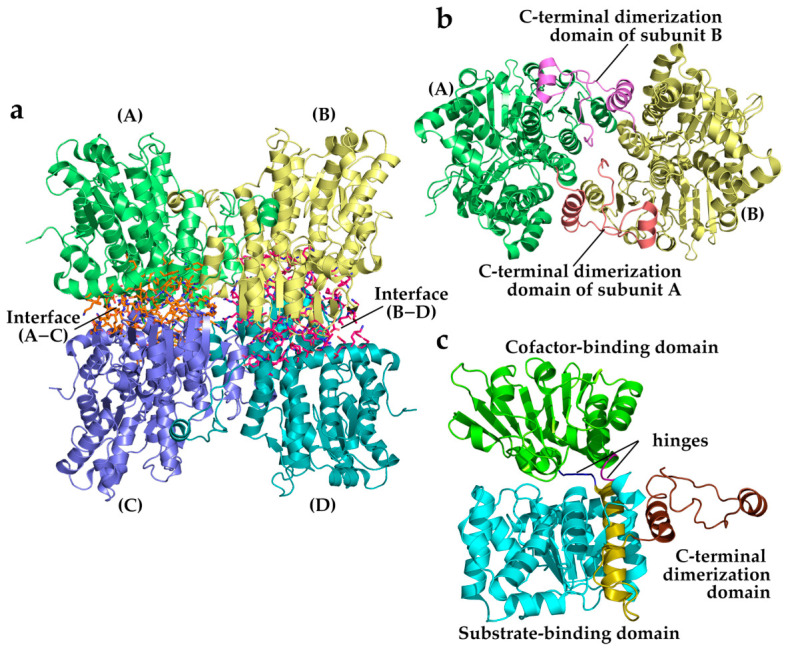
Quaternary and monomeric structure of SAHH from *B. elkanii* (PDB code 4LVC) [31]. (**a**) Tetrameric assembly viewed along the xy diagonal, with residues mediating inter-dimer contacts between the AB and CD dimers shown as sticks. Subunits A, B, C, and D are colored lime, yellow, slate, and teal, respectively. The interfaces of A–C and B–D are colored orange and pink, respectively. (**b**) The AB dimer, illustrating the reciprocal C-terminal domain swapping between subunits A (lime) and B (yellow) that stabilizes the intimate dimer interface. Their C-terminal dimerization domains are highlighted in salmon and violet. (**c**) Architecture of an individual subunit. The substrate-binding domain, cofactor-binding domain, and C-terminal dimerization domain are colored cyan and olive, chartreuse, and chocolate, respectively. The two hinges linking the substrate-binding and cofactor-binding domains are colored purple and blue, respectively. The molecular graphics were rendered using PyMOL 3.1.6.1.

The high degree of amino acid sequence conservation and the invariant nature of catalytic residues throughout evolution enable robust cross-species analysis of structure–function relationships [17]. Using SAHH from *Bradyrhizobium elkanii* (*Be*SAHH, PDB code 4LVC [29]) as an example, the quaternary organization can be described as a homotetramer with dihedral (D2) symmetry arranged along the xy diagonal. This homotetramer is assembled as a dimer-of-dimers [AB][CD] through an asymmetric oligomerization pattern mediated by a distinct interface between the AB and CD dimers [30] (Figure 3a).

As illustrated in Figure 3b, the C-terminal domain (residues L427–Y473) of subunit A extends into the catalytic region of the adjacent subunit B, and vice versa. This domain swapping of C-terminal tails between paired subunits stabilizes the two identical dimers (AB and CD). This association is primarily mediated by a network of hydrogen bonds and hydrophobic interactions between residues from the cofactor-binding domains and C-terminal domains of opposing subunits, as well as contacts between the swapped C-terminal regions, thereby creating a stable quaternary structure. Consequently, the tetramer is accurately described as a dimer of intimate dimers.

However, the homotetrameric assembly is not universally conserved. Most organisms, such as *B. elkanii*, possess SAHH complexes of approximately 200 kDa, consistent with a tetrameric assembly [29,31]. In contrast, *Ll*SAHH is a stable homodimer in solution, and it is currently the only plant SAHH experimentally confirmed to adopt a dimeric state [32]. A reduced number of inter-subunit hydrogen bonds (approximately two to three times fewer than non-plant orthologs) destabilizes the tetramer, and the enzyme functions predominantly as a homodimer [20]. Among bacteria, *Legionella pneumophila* SAHH (*Lp*SAHH) was the first bacterial enzyme found to be dimeric [10]. Similar to *Ll*SAHH, the dimer interface of *Lp*SAHH does not provide enough polar contacts to support tetramer formation. A broader overview of oligomeric state distribution across all three domains of life is presented in Section 6.

Despite these differences in oligomeric state, the core dimer architecture is strikingly conserved. Structural superposition of the AB dimers from the tetrameric *Be*SAHH (PDB code 4LVC) and the dimeric *Ll*SAHH (PDB code 3OND) yields a Cα RMSD of 1.34 Å over 928 aligned residues, with a pairwise sequence identity of approximately 61.3% (calculated using Clustal W). This high structural similarity indicates that the fundamental dimerization unit is a rigid and conserved evolutionary module and that the tetramer-to-dimer transition in plants is driven primarily by the loss of key inter-dimer contacts rather than by a rearrangement of the dimer itself [10].

As shown in Figure 3c, each SAHH subunit is organized into three distinct domains: a substrate-binding domain (residues M1–V221, cyan; residues M397–V426, olive), a cofactor-binding domain (residues Y234–G391, chartreuse), and a smaller C-terminal dimerization domain (residues L427–Y473, chocolate). Both the substrate-binding domain and cofactor-binding domain adopt variants of the Rossmann fold, a structural motif highly conserved across SAHH orthologs from different organisms [17]. The substrate-binding domain is divided into two regions (cyan and olive) by the cofactor-binding domain, and these domains are interconnected by two flexible hinge regions (residues N222–L233, purple; H392–V396, blue), which enable the cofactor-binding domain to rotate toward the substrate-binding domain and stabilize the tetramer during conformational changes [33].

### 4.2. Substrate-Binding Domain

**Figure 4:**
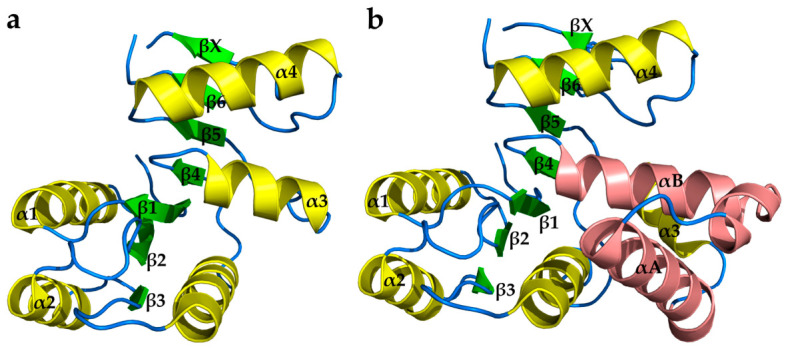
Two representative Rossmann fold topologies of the substrate-binding domain. (**a**) The compact fold lacking the ~40-residue insertion, exemplified by the archaeal *Sulfolobus acidocaldarius* SAHH (PDB code 7R39). This topology is also found in most eukaryotes (e.g., mammals, fungi) and some bacteria. (**b**) The topology containing the insertion (α-helices αA and αB, highlighted in salmon) exemplified by the bacterial *B. elkanii* SAHH (PDB code 4LVC). This topology is also characteristic of plant SAHHs and many other bacteria. α-helices, β-strands, and coils are colored yellow, green, and marine, respectively. The molecular graphics were rendered using PyMOL 3.1.6.1.

The substrate-binding domain is the largest domain, comprising more than 200 amino acid residues. As illustrated in Figure 4a, the substrate-binding domain exhibits a canonical Rossmann-fold topology, characterized by a central six-stranded parallel β-sheet (arranged in the order 3–2–1–4–5–6, green) flanked on both sides by α-helices (α1–α2 and α3–α4, yellow). An additional parallel strand, denoted βX, is also present.

Notably, SAHH from plants and many bacteria contains an insertion of approximately 40 amino acid residues, which folds into two atypical α-helices (αA and αB, salmon). This insertion forms a solvent-exposed structural element that modulates ligand accessibility and solvent interaction (Figure 4b) [20]. Moreover, the substrate-binding domain is located on the outer surface and plays little or no role in inter-subunit interactions. The substrate-binding pocket of SAHH is hydrophobic, accommodating the adenine moiety of the substrate (SAH) via hydrophobic interactions and hydrogen bonds. Meanwhile, the Hcy moiety is situated within a channel formed by conformational changes upon substrate binding [34].

### 4.3. Cofactor-Binding Domain

**Figure 5:**
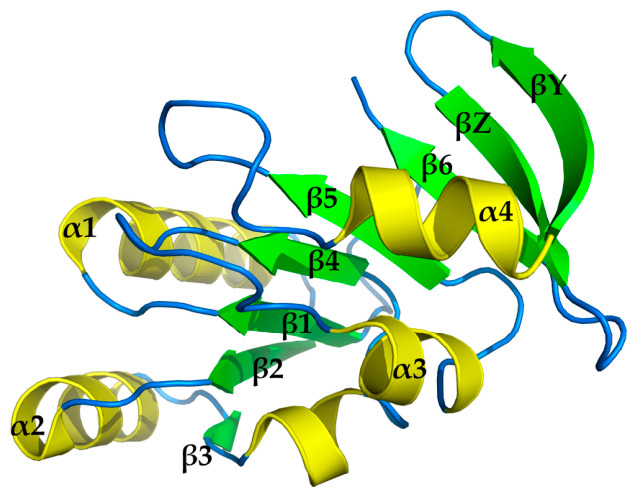
Rossmann fold topology of the cofactor-binding domain in bacterial *B. elkanii* SAHH (PDB code 4LVC). α-helices, β-sheets, and coils are shown in yellow, green, and marine, respectively. The molecular graphic was rendered using PyMOL 3.1.6.1.

The cofactor-binding domain is slightly smaller than the substrate-binding domain. It adopts a tertiary structure typical of nucleotide-binding proteins, featuring an extended Rossmann-like fold composed of eight β-strands [17] (Figure 5). Six of these (β1–β6, green) form a parallel sheet with topology 3–2–1–4–5–6, surrounded by α-helices (α1–α2 and α3–α4, yellow). Two additional short antiparallel strands, βY and βZ, are inserted between β5 and β6. This arrangement of β-sheets and surrounding α-helices creates the cofactor-binding pocket, enabling effective NAD^+^ binding and facilitating electron transfer during catalysis [16].

The cofactor-binding domain contains two conserved sequence motifs critical for NAD^+^ binding [35,36]. The first is a glycine-rich loop (GxGxxG, corresponding to residues 269–274 in *Ll*SAHH, PDB code 3OND) located between β1 and α1, with an aspartate residue at the N-terminus of α1 forming an electrostatic interaction with the β-phosphate group of NAD^+^. The second motif is an acidic residue (glutamate or aspartate) at the C-terminus of β2, which participates in hydrogen bonding with the 2′- and 3′-hydroxyl groups of the NAD^+^ ribose moiety. Given the rigidity of the NAD^+^-binding pocket, the cofactor-binding domain is less flexible than the substrate-binding domain [37].

### 4.4. C-Terminal Dimerization Domain

**Figure 6:**
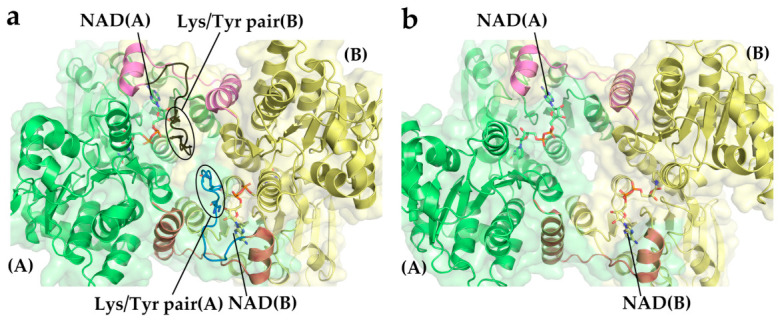
Visualization of the AB dimer interface illustrating the C-terminal dimerization domain in SAHH. (**a**) Eukaryotic and most bacterial SAHHs, exemplified by *Homo sapiens* SAHH (PDB code 3NJ4). The C-terminal tail of subunit A (salmon and marine, bottom) extends into the cofactor-binding domain of subunit B (violet and black, top), and vice versa. The conserved Lys/Tyr pair (K426 and Y430; black-circled region) at the tip of each tail anchors the NAD^+^ cofactor across the subunit interface. (**b**) Archaeal and certain bacterial SAHHs, exemplified by *T. maritima* SAHH (PDB code 3X2E). The C-terminal tails for subunits A and B are shown in salmon and violet, respectively. The C-terminal truncation eliminates the extended tail together with its Lys/Tyr pair (corresponding to the black-circled region in panel (**a**)), leaving the cofactor-binding pocket without inter-subunit anchoring of NAD^+^. Subunits A and B are depicted in lime and yellow, respectively, and displayed as surfaces with 75% transparency. The same view orientation and magnification are used in both panels to facilitate direct comparison. The molecular graphics were rendered using PyMOL 3.1.6.1.

The C-terminal region of SAHH plays a critical role in stabilizing the NAD^+^ cofactor, yet its length and mode of interaction with the cofactor vary significantly across species, with direct consequences for structural stability and catalytic efficiency [17]. In eukaryotic and many bacterial SAHHs, the C-terminal tail extends into the cofactor-binding pocket of an adjacent subunit, where a conserved Lys/Tyr pair (e.g., K426 and Y430 in human SAHH) anchors the cofactor by forming hydrogen bonds with the ribose and pyrophosphate moieties [28,37,38] (Figure 6a). The precise hydrogen-bonding network is detailed in Section 5.4.

The tail constitutes a small dimerization domain (<30 residues) that typically folds into a helix-loop-helix motif [34] and protrudes into the neighboring catalytic domain, establishing extensive inter-subunit interactions that stabilize the homotetrameric interface. Its terminal residues also directly participate in shaping the adenosine-binding pocket [16]. Moreover, the C-terminal tails, together with helices from the cofactor-binding domains of all subunits, assemble into a central channel that traverses the core of the tetramer [33]. Although not directly involved in catalysis, this flexible hub is lined by 13 highly conserved hydrophilic residues that support hydration or salt-bridge networks, thereby accommodating cooperative and reversible conformational rearrangements during the open-to-closed transition and preserving tetramer integrity [37].

In contrast, SAHHs from archaea and certain bacteria have undergone a C-terminal truncation of 8–11 residues (Figure 6b and Figure S1) that eliminates both the independent dimerization domain and the conserved Lys/Tyr pair, forcing these enzymes to oligomerize via a mechanism distinct from C-terminal domain swapping [35]. This architectural simplification is directly linked to thermophilic adaptation: removal of the flexible C-terminal tail enhances protein rigidity, thereby increasing thermostability at the expense of the inter-subunit NAD^+^ clamp. Consequently, NAD^+^ affinity decreases by approximately 280-fold; however, this reduction is partially compensated by the elevated intracellular NAD^+^ pools characteristic of hyperthermophilic growth conditions [1]. Despite lacking these key hydrogen-bonding residues, these truncated enzymes still bind NAD^+^ at a stoichiometry of one NAD^+^ per monomer, implying alternative stabilization strategies, such as aromatic and hydrophobic interactions, are deployed.

Phylogenetic analyses suggest the elongated C-terminus conserved in most bacteria and eukaryotes is an adaptive trait acquired to reinforce quaternary stability, particularly under moderate conditions [38]. Thus, the length and interaction pattern of the C-terminal domain fundamentally govern both the mechanism of NAD^+^ anchorage and tetramer integrity. The pronounced divergence among archaeal, bacterial and eukaryotic SAHHs in this region reflects distinct evolutionary trajectories that fine-tune catalytic efficiency and thermostability, while also presenting species-specific structural features exploitable in drug design.

## 5. Interaction with Other Molecules

### 5.1. Molecular Gate

**Figure 7:**
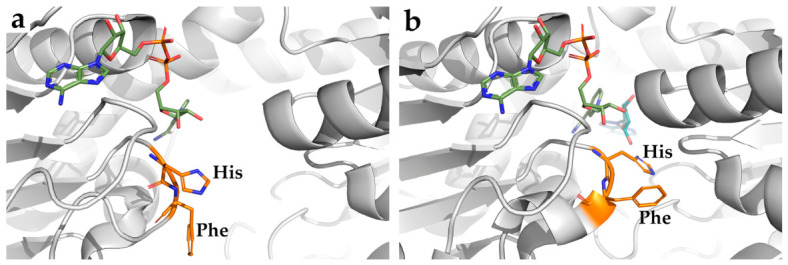
Conserved HF dyad functions as a molecular gate regulating active site accessibility. Model based on the structure of *B. elkanii* SAHH (PDB code 4LVC) [31]. (**a**) Substrate-free SAHH with the solvent channel open and the binding site accessible (chain D). (**b**) SAHH in complex with adenosine, showing the fully closed conformation (chains A, B, C). The gating motif (HF dyad) is highlighted in orange. The cofactor (NAD^+^) and ligand (adenosine) are highlighted in smudge and deep teal, respectively. The molecular graphics were rendered using PyMOL 3.1.6.1.

SAHH undergoes substantial conformational changes upon substrate binding. Structural comparisons between the open (NAD^+^-bound) and the closed (NAD^+^- and adenosine-bound) states reveal a pronounced closure of the interdomain cleft within each subunit [31,39]. Central to this switch is a solvent-accessible channel gated by the His-Phe dyad of the highly conserved GHF motif [30], exemplified by H301-F302 in human and mouse SAHH, H363-F364 in *M. tuberculosis* SAHH [40], and H350-F351 in plant SAHH [20]. The peptide bond between the Cα atom of histidine and phenylalanine undergoes a 180° flip and functions as a molecular gate, with the glycine of the motif providing the necessary hinge flexibility [37,40]. In the substrate-free state (Figure 7a), the histidine side chain adopts a “flipped-out” conformation that opens the channel, allowing the Hcy moiety of SAH to access the active site [40]. Upon binding of adenosine or its analogs (Figure 7b), the histidine flips to the “flipped-in” state, closing the channel, sealing the active site from solvent, and creating an enclosed environment for the stability of the enzyme–adenine complex, even protecting adenine from dissociation during prolonged dialysis [19,41].

Crystal structures from mammalian, mycobacterial, and plant enzymes directly support this conserved gating mechanism [20,37,40]. Notably, in the mouse SAHH–adenosine complex, His301 simultaneously occupies both “flipped-in” and “flipped-out” conformations, revealing the dynamic flexibility required for product release [37]. In *M. tuberculosis* SAHH, the gate must adopt the “flipped-out” state to accommodate the Hcy tail and reverts to the “flipped-in” state after its cleavage [40]. This universal substrate-access switch is also confirmed in archaeal SAHHs, where greater structural flexibility and thermal mobility of thermophilic and archaeal SAHHs may facilitate gating dynamics [1].

Despite often being described as a substrate-triggered switch coupled to domain closure, the gate exhibits more complex behavior. The glycine hinge of the GHF motif permits gating motions that are not strictly slaved to the global open-to-closed transition [37]. In *Be*SAHH, the gate status does not simply correlate with the overall subunit conformation or active-site occupancy; even with the same ligands, the gate can adopt different states [29]. Such non-synchronous behavior demonstrates that interdomain cleft closure and gate conformation can sample semi-independent states, introducing conformational heterogeneity. Moreover, metal ions can directly commandeer the gate. In *Pseudomonas aeruginosa* SAHH (*Pa*SAHH), Zn^2+^ ions coordinate with H323 in the GHF motif, locking the gate in the closed position and trapping the enzyme in an inactive conformation [42].

The molecular gate is conserved and ensures the opening and closing of the active site in all SAHH-catalyzed reactions. However, the specific regulatory mechanisms, such as the stability of dual conformations, and the physicochemical properties of the channel, including size and hydrophobicity, vary among different species. These variations constitute species-specific targets that must be considered when designing inhibitors directed at the gating apparatus. From an evolutionary perspective, the enhanced conformational flexibility observed in the gate of thermophilic and archaeal SAHHs may facilitate catalysis at elevated temperatures, where intrinsically faster molecular motions demand efficient active-site access and product release [1]. Beyond intrinsic conformational dynamics, the gate region also harbors a conserved metal-binding site that directly modulates enzyme activity and stability.

### 5.2. Regulation of SAHH by Metal Ions

Metal ion coordination is a widespread mechanism for regulating enzymatic stability and catalytic activity [43]. In SAHH, the hinge region that houses the His-Phe gate also provides a conserved coordination site for cations, as detailed below.

#### 5.2.1. Structural Basis of Cation Coordination

SAHH is a metal-binding protein, with cations such as Na^+^, K^+^, Zn^2+^, and Cu^2+^ frequently observed in its crystal structures, often depending on crystallization conditions [1,44,45,46]. These cations typically bind within a dedicated loop proximal to the purine ring of the ligand without directly interacting with the substrate itself [17,42]. This coordination loop forms part of the hinge region connecting the substrate-binding and cofactor-binding domains and is implicated in the substrate-induced domain movements essential for catalysis [20,33]. During the catalytic cycle, the bound cation induces conformational changes in the active site or adjacent regulatory regions by forming coordination bonds with surrounding amino acids, thereby modulating substrate affinity and structural dynamics [47]. For instance, mutation of a conserved histidine within this hinge region—also involved in the “molecular gate” mechanism—disrupts domain motion and significantly impairs enzymatic activity [48].

**Figure 8:**
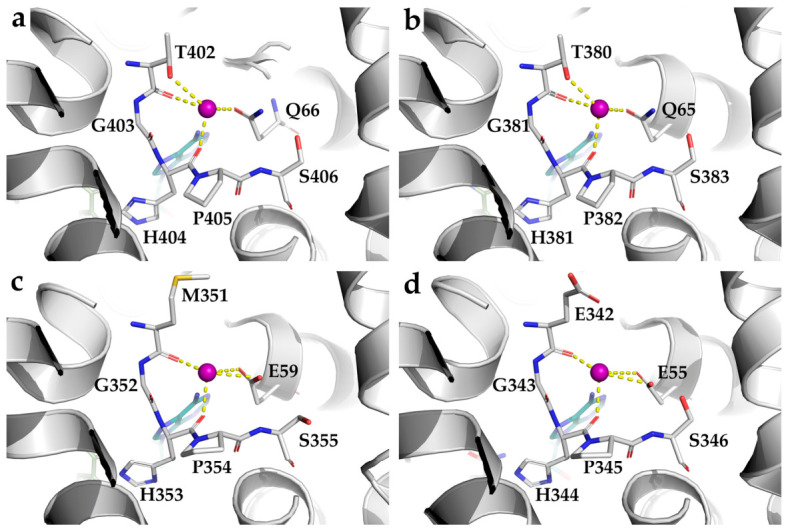
Cation recognition in SAHH from (**a**) plant (*L. luteus*, PDB code 3OND), (**b**) bacteria (*P. aeruginosa*, PDB code 6F3M), (**c**) animal (*H. sapiens*, PDB code 3NJ4), and (**d**) archaeon (*S. acidocaldarius*, PDB code 7R39). The coordination interactions are indicated by yellow dashed lines. Metal cations are depicted as purple spheres. The molecular graphics were rendered using PyMOL 3.1.6.1.

As illustrated in Figure 8, metal cations are coordinated in a slightly distorted pentagonal bipyramidal geometry. In plant SAHH (e.g., *Ll*SAHH, Figure 8a) [20], the coordination interaction network comprises two main-chain carbonyl oxygens from the conserved TGHPS loop, the side-chain oxygen of a threonine (T402), and the amide oxygen of a glutamine (Q66), as seen in most bacterial SAHHs (e.g., *Pa*SAHH, Figure 8b) [42]. This glutamine is highly conserved in most fungi and bacteria but is substituted by glutamate (e.g., E59 in *H. sapiens* SAHH (*Hs*SAHH), Figure 8c; E55 in *S. acidocaldarius* SAHH (*Sa*SAHH), Figure 8d) in mammals and certain protozoa [1]. In archaea, this position can be occupied by glutamine, glutamate, or an unprotonated lysine acting as a hydrogen-bond acceptor (Figure S1) [35].

#### 5.2.2. Effects of Metal Ions on Activity and Stability

The influence of alkali metal ions (K^+^, Na^+^, Rb^+^, Cs^+^, Li^+^) on SAHH depends on ionic radius and coordination number [42]. Complex stability decreases with increasing ionic radius (Li^+^: 0.76 Å < Na^+^:1.02 or 1.12 Å < K^+^: 1.46 Å < Rb^+^:1.56 Å < Cs^+^: 1.67 Å), as larger ions cannot be fully accommodated within the binding pocket. Smaller ions such as Li^+^ interact only weakly with the coordinating glutamine. Optimal binding and activation are observed with K^+^, which exhibits an ideal balance of radius and coordination number. Higher coordination numbers can impede complex dissociation, trapping the enzyme in a closed, inactive conformation. Transition metal ions (e.g., Fe^2+^, Ni^2+^, Co^2+^, Cu^+^, Cu^2+^, Zn^2+^, Cd^2+^, Hg^2+^) generally act as inhibitors [46]. For *Pa*SAHH, Cd^2+^, Zn^2+^, and Hg^2+^ bind competitively at the cation site, while Cu^2+^ inhibits non-competitively by binding in the central channel, disrupting intersubunit interactions and the NAD^+^-binding network, leading to cofactor release and loss of activity. These insights into metal-dependent regulation provide a molecular framework for understanding how cation availability modulates SAHH function and inform the design of cation-mimetic inhibitors.

### 5.3. Interactions with the Substrate

**Figure 9:**
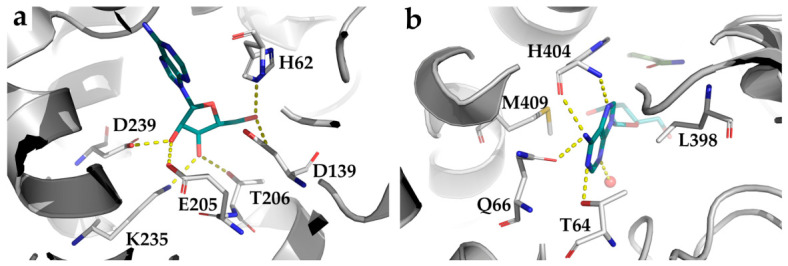
Interactions between the (**a**) sugar moiety and (**b**) adenine moiety of adenosine and conserved residues in SAHH, exemplified by plant SAHH from *L. luteus* (PDB code 3OND). The water atom is depicted as a red sphere. The ligand, adenosine, is depicted as deep teal sticks. Hydrogen bonds are indicated by yellow dashed lines. The molecular graphics were rendered using PyMOL 3.1.6.1.

The binding mode of nucleoside substrates is highly conserved across SAHHs from diverse phylogenetic lineages, with specific residues in both the substrate-binding domain and cofactor-binding domain participating in ligand recognition [33,34,49]. Comparative structural analysis reveals a consistent hydrogen-bonding network between the D-ribose and adenine moiety of adenosine and conserved amino acids from plants, bacteria, animals, and archaea (Figure 9; Table S1).

#### 5.3.1. Interaction with the Substrate Sugar Moiety

The substrate ribose moiety forms a hydrogen-bonding network with conserved residues through three hydroxyl groups (Figure 9a; Table S1). The 2′-OH forms bidentate hydrogen bonds with the ε-carboxylate of a glutamate at the C-terminus of strand β5 in the substrate-binding domain and the δ-carboxylate of an aspartate within a loop following strand β6 in the cofactor-binding domain. The 3′-OH is stabilized by a threonine in the loop connecting β5 and α4 and a lysine at the C-terminus of the helix preceding β6. The 5′-OH stabilizes the substrate prior to catalysis. It participates in hydrogen bonds with the δ-carboxylate of an aspartate at the C-terminus of β4 and the Nε2 atom of a histidine in the loop following β1. In the absence of a ribose-containing ligand, these sites are often occupied by water molecules (e.g., in *Ll*SAHH–adenine) [20] or a phosphate anion (e.g., in *Pa*SAHH–adenine) [17], which mimic the ribose-hydroxyl hydrogen-bonding pattern through the same conserved residues.

The ribose ring puckering is inherently flexible and differs markedly across species, a variation primarily determined by the presence or absence of an approximately 40-amino-acid insertion within the substrate-binding domain. SAHHs containing this segment (e.g., from plants and certain bacteria) preferentially adopt a C4′-exo ribose conformation, whereas mammalian enzymes lacking this insert maintain a C4′-endo pucker. This conformational preference arises from the insert reshaping the local hydrogen-bonding network and steric environment of the active site, thereby indirectly modulating ribose positioning [17]. In *M. tuberculosis* SAHH, the insertion deepens the substrate-binding pocket, a species-specific structural feature that not only contributes to functional specialization but also provides a structural basis for the design of selective anti-tuberculosis inhibitors [40]. The ability of the insertion to stabilize the C4′-exo pucker is therefore best understood as an adaptive trait that fine-tunes substrate-channel geometry for optimal hydride transfer in organisms with distinct metabolic demands or cellular redox environments [20,45,50].

#### 5.3.2. Interaction with the Substrate Adenine Moiety

The purine ring is nestled within a hydrophobic crevice formed by conserved side-chain and backbone atoms (Figure 9b; Table S1) and is stabilized by an intricate network of hydrogen bonding, C–H···π interactions, and hydrophobic contacts [20].

The correct *anti*-conformation and planarity of the adenine ring are locked through a precise multi-point hydrogen-bonding network (Figure 9b; Table S1). The exocyclic N6 amino group forms bidentate hydrogen bonds with the main-chain carbonyl oxygen of a conserved histidine from the GHF motif and with the side-chain carbonyl oxygen of a glutamine or glutamic acid that defines the substrate-specificity fingerprint motif (HxTxQ(E) or HxExK) [1]. This dual recognition not only orients the purine ring correctly but also contributes significantly to binding affinity [20]. The same histidine accepts a hydrogen bond from the heterocyclic N7 atom via its main-chain amide nitrogen, while N1 is anchored by the hydroxyl group (Oγ1) of a conserved threonine; together, these interactions maintain the ring planarity [20,40]. In addition, the N3 atom frequently interacts with a structural water molecule, providing further stabilization [51].

Complementing this polar network, both faces of the purine ring are sandwiched by the side chains of conserved leucine and methionine (Figure 9b; Table S1). Through hydrophobic interactions and C–H···π interactions, these residues form a hydrophobic cage that greatly enhances binding stability and clamps the base in place [37]. Notably, these leucine and methionine residues lie on adjacent α-helices connected by a flexible hinge, a region directly involved in the open-to-closed conformational transition during catalysis.

This integrated anchoring system is exceptionally conserved. Disruption of these interactions, for instance, through inhibitor modifications that interfere with the polarity recognition or the hydrophobic cage, is an effective strategy for abrogating enzyme activity. The mutually exclusive distribution of the fingerprint motif across species with HxTxQ(E) or HxExK indicates a single evolutionary transition event that accompanied the divergence of these major archaeal lineages and their adaptation to distinct ecological niches [1].

### 5.4. Interactions with the Cofactor NAD^+^

**Figure 10:**
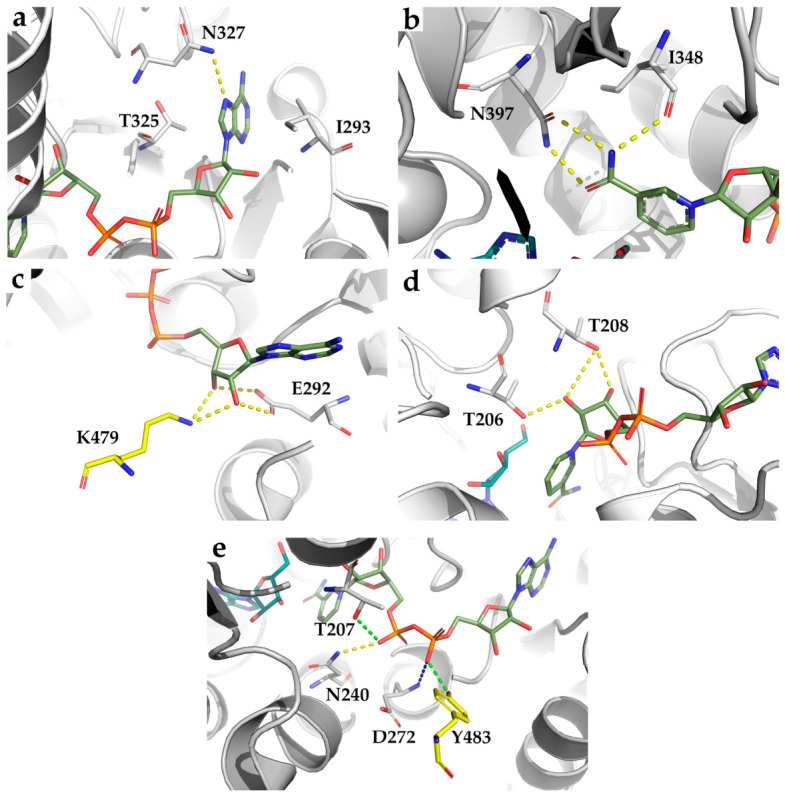
Recognition mode of the cofactor NAD^+^ exemplified by SAHH from the plant *L. luteus* (PDB code 3OND). (**a**) Interaction with the NAD^+^ adenine moiety; (**b**) interaction with the NAD^+^ nicotinamide moiety; (**c**) interaction with the NAD^+^ adenosine ribofuranose moiety; (**d**) interaction with the NAD^+^ nicotinamide ribofuranose moiety; (**e**) Interaction with the NAD^+^ phosphate groups. The cofactor NAD^+^ is depicted as smudge sticks. Residues from the neighboring subunit are shown as yellow sticks. Yellow dashed lines indicate hydrogen bonds; the blue dashed line indicates electrostatic interactions; green dashed lines indicate polar interactions. The molecular graphics were rendered using PyMOL 3.1.6.1.

The catalysis of SAHH depends on a tightly bound cofactor NAD^+^, which is stabilized through an extensive network of polar and nonpolar interactions involving its adenine, nicotinamide, D-ribose sugars, and pyrophosphate moiety [20,39,42]. Therefore, elucidating the functional roles of these conserved residues is key to understanding how the enzyme integrates robust NAD^+^ binding with regulable catalysis (Figure 10; Table S1).

#### 5.4.1. Interaction with the Cofactor Adenine Moiety

Recognition of the adenine moiety of NAD^+^ is highly conserved across species and relies on hydrophobic stacking and a polar interaction network to precisely anchor the base (Figure 10a; Table S1). The purine ring is accommodated within a hydrophobic cavity formed by the side chains of hydrophobic residues, typically an isoleucine or valine in the loop between β2 and α1 and a threonine in the loop connecting β4 and α3. Through hydrophobic contacts and C–H···π interactions, these residues create a hydrophobic sandwich that stabilizes the planarity of the ring [20].

Furthermore, a conserved asparagine within the β4–α3 loop provides a specific hydrogen bond between its side-chain amide nitrogen (Nδ2) and the N7 atom of adenine (Figure 10a; Table S1). This polar contact locks the base in the proper *anti* conformation, which is essential for subsequent interactions with the C-terminal tail of the adjacent subunit [20,52].

Notably, species-specific variations fine-tune the cofactor-binding interface. In the SAHH from thermophilic archaeon *Sa*SAHH, the hydrophobic residue (isoleucine or valine) is replaced by a hydrophilic serine (S235) [1], attenuating hydrophobic interactions. In *Hs*SAHH, however, the asparagine is substituted by cysteine (C278) with loss of the N7 hydrogen bond [10], reflecting an evolutionary fine-tuning of the cofactor-binding interface.

#### 5.4.2. Interactions with the NAD^+^ Nicotinamide Moiety

The nicotinamide moiety is accommodated within a narrow cavity flanked by a highly conserved Cys–Val pair (or rare variants such as Thr–Val or Thr–Cys in SAHHs from *Mycobacterial* and *T. maritima*, respectively) [12,40] and the D-ribose ring of the substrate on the opposite side [20,34]. This structure ensures precise alignment of the nicotinamide C4 atom with the substrate ribose C3′ for catalysis.

The amide group (N7 and O7) of the nicotinamide ring is recognized through bidentate hydrogen-bonding interactions with a conserved asparagine residue (Figure 10b; Table S1). Its side-chain Oδ1 atom accepts a hydrogen bond from N7, while its Nδ2 atom donates one to O7. Additionally, the N7 atom forms an additional hydrogen bond with the main-chain carbonyl oxygen of an isoleucine in most species or a serine in *Sa*SAHH (Table S1). This multidentate polar network firmly locks the nicotinamide ring in a defined orientation.

Importantly, this recognition system possesses inherent conformational dynamics. In the ligand-free open conformation, although the Asn-mediated bidentate interaction may be partially retained, the nicotinamide ring preserves rotational freedom around the N-glycosidic bond. Upon substrate binding, the closed conformation is induced, and the substrate ribose ring serves as the opposing cavity wall to complete the final rigid lock [17]. This dynamic property is central to the open-to-closed conformational transition during the catalytic cycle, ensuring precise alignment of catalytic groups for efficient hydride transfer [16,37].

#### 5.4.3. Recognition Mode of the NAD^+^ Ribofuranose

NAD^+^ contains two distinct ribofuranose rings: one linked to the adenine (adenosine ribose) and the other to the nicotinamide (nicotinamide ribose). These sugar rings adopt significantly different puckering conformations in both the open and closed enzyme states. The adenosine ribose predominantly adopts an O4′-*endo* or C1′-*exo* puckering, whereas the nicotinamide ribose favors a C2′-*endo* puckering.

**Adenosine ribose recognition.**

A conserved acidic residue (glutamate) at the C-terminus of strand β2 orients its carboxylate group (Oε) inward to form bidentate hydrogen bonds with both the 2′- and 3′-hydroxyl groups of the adenosine ribose (Figure 10c; Table S1) [17]. This interaction constitutes the primary conformational lock and represents a canonical ribose-recognition mode of Rossmann-fold enzymes [17,39].

Furthermore, a lysine from the C-terminal domain of an adjacent subunit provides an additional anchor. Its Nζ atom forms bifurcated hydrogen bonds with the same 2′- and 3′- groups (Figure 10c; Table S1), firmly securing ribose in the binding pocket [38]. While the K426A/E mutations in human SAHH disrupt NAD^+^ binding, promote monomerization, and inactivate the enzyme, demonstrating the indispensability of this intersubunit stabilization [53].

In contrast, archaeal SAHHs (e.g., *Sa*SAHH) lack this lysine due to a characteristic C-terminal truncation (Table S1) [17]. NAD^+^ binding therefore depends solely on the conserved glutamate and compensatory main-chain interactions, resulting in a drastic drop in affinity [39,49]. However, the loss of this molecular clamp loosens the intersubunit interface, paradoxically endowing the enzyme with the enhanced structural rigidity and thermal stability required for high-temperature environments, representing a classic evolutionary trade-off between catalytic efficiency and structural stability [1].

**Nicotinamide ribose recognition.**

Recognition of nicotinamide ribose is primarily mediated by two conserved threonine residues located at the N-terminus of helix α1 in the cofactor-binding domain (Figure 10d; Table S1). The Oγ1 atoms of both threonines form hydrogen bonds with the 2′-OH group of the ribose. In most species, the Oγ1 atom of the second threonine (e.g., T208 in *Ll*SAHH) is equidistant (~3.3 Å) from both the 2′- and 3′-OH groups, enabling bifurcated hydrogen bonding that locks the nicotinamide ribose in a C2′-endo conformation. This precise geometric constraint positions the C4 atom of the nicotinamide ring directly opposite the substrate ribose C3′, a prerequisite for efficient hydride transfer [17].

A notable exception occurs in some SAHHs from extremophilic microorganisms, such as *T. maritima* SAHH (*Tm*SAHH, PDB code 3X2E), where the Oγ1 of the homologous threonine (T141) is located ~4.3 Å from the 2′-OH, precluding hydrogen bond formation and resulting in the loss of this bifurcated recognition [24,54]. This structural divergence arises from a C-terminal truncation characteristic of archaeal/thermophilic SAHHs, which reshapes the geometry of the nicotinamide ribose-binding pocket [1]. In *Tm*SAHH, compensatory interactions, such as hydrogen bonds between E227 and the ribose hydroxyls, partially restore the polar contact network, exemplifying an evolutionary trade-off between cofactor affinity and the structural rigidity required for high-temperature adaptation [39].

#### 5.4.4. Interaction of the Cofactor Phosphate Groups

The α-phosphate group (Oα1) engages in an electrostatic interaction with the main-chain amide nitrogen of the fourth residue within the glycine-rich loop (GxGxxG), typically an aspartate or tryptophan residue (Figure 10e; Table S1), a canonical mode of pyrophosphate recognition in Rossmann-fold enzymes [55,56]. The β-phosphate is further anchored by a polar interaction with the side-chain hydroxyl (Oγ1) of a threonine at the N-terminus of helix α4 [17]. In eukaryotic and bacterial SAHHs, an additional hydrogen bond to the phosphate groups is supplied by the side-chain Nδ2 atom of an asparagine in the loop following strand β6. In human SAHH, however, this interaction is uniquely mediated by the side-chain oxygen (Oδ2) of the same asparagine, representing a subtle intraspecies variation [39].

The integrity of this phosphate-binding network depends on tetramer assembly. As seen in Table S1, in eukaryotic and bacterial SAHHs, a highly conserved tyrosine from the C-terminal domain of the neighboring subunit inserts its phenolic hydroxyl into the active site, forming an inter-subunit polar interaction with the α-phosphate that enhances cofactor affinity [49]. Archaeal SAHHs, as well as some thermophilic enzymes, by contrast, lack this tyrosine entirely owing to a characteristic C-terminal truncation, resulting in the loss of this inter-subunit anchoring point and accounting for the several-orders-of-magnitude lower NAD^+^ affinity compared to their eukaryotic/bacterial counterparts [1,17]. Together with the absence of the adenine-N7 hydrogen bond in some lineages (Section 5.4.1), this systematic reduction of the cofactor-binding network in archaeal and some thermophilic SAHHs does not represent a defective state but rather an evolutionary optimization that shifts the enzyme from a high-affinity/low-stability regime to a lower-affinity/high-stability regime suitable for hyperthermophilic function.

Notably, this reduction in cofactor affinity coincides with the HxExK motif switch that redirects substrate preference from SAH to SIH in euryarchaeal enzymes (Section 2.2 and Section 5.3.2). The concomitant weakening of both cofactor binding and substrate anchoring suggests a coordinated relaxation of the enzyme’s catalytic constraints, which, together with the enhanced structural rigidity conferred by the C-terminal truncation, enables efficient catalysis under the extreme temperature and altered metabolic conditions of hyperthermophilic habitats.

#### 5.4.5. Functional Effects of Exogenous NAD^+^ on Activity and Stability

The catalytic cycle of SAHH is intrinsically linked to the redox transition of a tightly bound NAD(H), which remains associated with the enzyme throughout turnover and does not require exogenous cofactor addition under physiological conditions [57]. However, preincubation with supplemental NAD^+^ has been shown to enhance both activity and structural stability across various SAHH orthologs. For instance, exogenous NAD^+^ activates *Dictyostelium discoideum* SAHH and stabilizes its tetrameric assembly by reinforcing intersubunit interactions [58]. Similarly, Ryuichi Iwasaki et al. [59] reported that NAD^+^ supplementation significantly increased the specific activity of recombinant SAHH by 1.7-fold at 60 °C. A notable strategy for sustaining high NAD^+^ levels was employed by Qian et al. [60], who co-expressed *Tm*SAHH and lactate dehydrogenase in *E. coli* to establish an efficient NAD^+^ regeneration system. Under elevated NAD^+^ concentrations (8 mM), the optimal reaction profile of *Tm*SAHH shifted to 100 °C (+15 °C) and pH 11.2 (+3.2 pH units), while specific activity increased nearly 6-fold (from 6.2 to 36.8 U/mg). The stabilizing effect of NAD^+^ is further corroborated by biophysical evidence: surface hydrophobicity decreased by 38.7% and 41.0% at 0.5 mM and 5 mM NAD^+^, respectively, indicating a more compact and thermostable conformation. Although these observations consistently support a role for NAD^+^ in enhancing structural integrity and catalytic performance, the precise molecular mechanism remains incompletely elucidated and warrants further investigation.

## 6. Biological Distribution and Sequence Analysis

SAHH is universally distributed across archaea, eukaryotes, and bacteria [16]. Analysis of available three-dimensional structures retrieved from the RCSB Protein Data Bank reveals that SAHHs typically function as oligomers with single-chain molecular weights ranging from 46 to 56 KDa. These enzymes exhibit lineage-specific variations in both sequence length and quaternary assembly (Table 1).

Archaeal SAHHs and those from certain bacteria (e.g., *Synechocystis* sp. PCC 6803 [1] and *T. maritima* [35]) generally lack 8–11 residues at the C-terminus (Figure S1), which precludes the inter-subunit domain swapping essential for C-terminal dimerization. An additional major structural variable is an insertion of approximately 40 residues near the substrate channel, present in many bacteria (e.g., *B. elkanii*, *Brucella abortus* 2308, *Burkholderia pseudomallei* 1710b, and *M. tuberculosis*) and plants (e.g., *L. luteus*), but absent in fungi, insects, and vertebrates [29,32,38,40]. Phylogenetic mapping indicates that this insertion represents a convergent evolutionary solution for modulating substrate-binding pocket depth and ribose puckering preference [10,20].

A complete species-by-species overview of quaternary structures is provided in Table 1. In eukaryotes, the enzyme is overwhelmingly homotetrameric [1,33,44,50,61,62,63]. The only experimentally confirmed exception among eukaryotes is *L. luteus*, whose SAHH functions as a stable homodimer in solution because of a weakened inter-dimer interface with two to three times fewer hydrogen bonds than non-plant orthologs (discussed in Section 4.1) [20]. Notably, other eukaryotes, such as *Leishmania major*, possess SAHH complexes consistent with a tetrameric assembly [63], indicating that the dimeric form is a derived trait specific to certain plant lineages rather than a universal eukaryotic characteristic.

In archaea, the oligomeric state is tetrameric across all structurally characterized orthologs [1]. The C-terminal truncation characteristic of these enzymes eliminates the Lys/Tyr pair that mediates inter-subunit NAD^+^ coordination in eukaryotic and most bacterial enzymes, yet they still assemble into functional tetramers via alternative stabilization strategies, such as aromatic and hydrophobic interactions [1].

Among bacteria, homotetramers are overwhelmingly dominant (Table 1). Two notable exceptions exist: *Lp*SAHH is the first identified bacterial SAHH that functions as a homodimer in its active state, a feature discussed in detail in Section 4.1 [10]; *Synechocystis* sp. SAHH has been characterized in both homodimeric and homotetrameric states; the solution depends on the protein concentration [36], demonstrating that oligomeric plasticity can occur even within a single species.

The conserved tetrameric architecture, prevalent in most eukaryotic and bacterial SAHHs, is considered the ancestral and functionally optimal state, ensuring high catalytic efficiency and structural stability [28,45,50,55]. Beyond these static lineage-specific adaptations, the oligomeric state can also be dynamically modulated. Post-translational modifications (e.g., O-GlcNAcylation, which promotes tetramer formation in human SAHH [68]) and cofactor concentration [59] influence the assembly state, fine-tuning activity in response to cellular cues. Mutations that disrupt tetramer formation (e.g., the K426E mutation in human SAHH) render the enzyme inactive by favoring monomer formation, underscoring the functional importance of correct oligomerization [53].

Collectively, the sequence and structural diversity of SAHH across archaea, bacteria, and eukaryotes illustrates a continuous evolutionary trade-off among catalytic efficiency, thermostability, and regulatory capacity. Three lineage-specific innovations underpin this functional divergence: (i) C-terminal truncation, which enhances protein rigidity at the expense of high-affinity NAD^+^ binding, thereby adapting enzymes to hyperthermophilic environments [39,69]; (ii) the independent acquisition of a 40-residue insertion in plants and certain bacteria that remodels the substrate-binding pocket and ribose puckering [20]; and (iii) the HxExK motif switch that rewires substrate specificity from SAH to SIH [1,40]. These adaptations not only clarify the metabolic logic of archaeal one-carbon metabolism but also offer unique structural targets for therapeutic intervention.

## 7. Applications in Medicine and Biotechnology

The structural and mechanistic principles elaborated in the preceding sections, including the conserved catalytic residues, cofactor dynamics, oligomeric plasticity, and metal-ion regulation, directly enable the following translational applications. Lineage-specific variations in substrate preference (SAH versus SIH [1,24]), quaternary assembly [10], and sensitivity to metal ions and cofactors [46,59] illustrate how catalytic performance can be tuned through structural modulation. These insights guide species-selective inhibitor design (Section 7.1); the coupling between cofactor occupancy and conformational changes underpins SAHH-based biosensing (Section 7.2); and the thermostability and efficient NAD^+^ cycling of engineered variants enable industrial biocatalysis (Section 7.3). Thus, fundamental knowledge of cofactor dynamics and molecular architecture provides a unified foundation for drug discovery [70], clinical diagnostics [71], and biomanufacturing [8,59].

### 7.1. Therapeutic Target and Drug Development

SAHH is a clinically attractive target because its inhibition elevates intracellular SAH, which in turn inhibits SAM-dependent methyltransferases essential for viral mRNA cap formation, epigenetic regulation and tumor-cell proliferation [72,73]. Despite decades of effort, no SAHH inhibitor has been clinically approved, exposing a persistent gap between potent preclinical activity and systemic toxicity.

Several structurally diverse inhibitors have been reported. The broad-spectrum antiviral MSD-914 potently inhibited filoviruses in vitro and fully protected mice, yet failed to improve survival in rhesus macaques, revealing a species-dependent efficacy barrier [74]. The reversible inhibitor DZ2002 ameliorated osteoarthritis, diabetic wound healing and dry eye disease in rodents by modulating MEK/ERK, MLL1/H3K4me3 and STAT3–PI3K–Akt–NF-κB pathways [75,76,77]. The natural product asarinin reverses ferroptosis resistance in lung cancer by inhibiting AHCY and sensitizing cells to the relevant treatment [78]. Hierarchical virtual screening combined with MD simulations identified compound 7 as a selective *M. tuberculosis* SAHH inhibitor devoid of Gram-negative toxicity [12]. In an anti-parasitic screen against *N. fowleri* SAHH, a lead compound with a binding energy of −11.4 kcal/mol was validated using 100 ns MD simulations and MM/GBSA [11]. 6′-β-fluoro-homoaristeromycin suppresses chikungunya virus replication [79]. Beyond classical methyl-cycle inhibition, a moonlighting function of AHCY has been uncovered: the AHCY–adenosine complex acts as a scaffold to inhibit the m^6^A demethylase FTO, thereby promoting fatty acid synthesis and tumorigenesis [80]. These discoveries extend the therapeutic concept beyond simple methyl-cycle disruption. Beyond therapeutics, SAHH has also been harnessed as a versatile biotechnological tool, with applications in enzymatic synthesis and biosensing that are discussed in Section 7.3.

Systemic toxicity remains the principal barrier to clinical translation. Pharmacological SAHH inhibition provokes endothelial senescence [81], exacerbates podocyte injury in diabetic nephropathy [82], and causes hepatotoxicity—triptolide’s liver toxicity is mediated through direct AHCY binding [83]. The active site is highly conserved, making selective targeting of pathogen SAHH over the human enzyme extremely difficult [40]. Consequently, classic adenosine analogs such as neplanocin A and 3-deazaneplanocin A (DZNep), despite potent in vitro and in vivo activity, have been abandoned or stalled owing to unacceptable toxicity [84,85]. As of mid-2026, no SAHH inhibitor has entered clinical trials; DZ2002 and MSD-914 remain at the preclinical stage. Indirect clinical validation comes from MAT2A inhibitor AG-270 and DGKζ inhibitor ASP1570, which target nodes flanking SAHH and have advanced to Phase I/II trials. The development status of representative SAHH inhibitors is summarized in Table 2; consistent with the challenges discussed above, none has progressed beyond preclinical evaluation.

Overcoming the toxicity barrier will likely require a multi-pronged strategy. First, structure-guided allosteric inhibitors targeting less conserved pockets could avoid cross-inhibition of host SAHH. Second, prodrugs or targeted delivery systems (e.g., peptide-conjugated DZ2002 [86]) may confine drug activity to diseased tissues or pathogens. Third, integrating SAH/SAM ratio monitoring as a pharmacodynamic biomarker could enable personalized dosing windows that balance antiviral or antitumor efficacy with systemic toxicity [87,88]. Combination regimens with ferroptosis inducers or immune checkpoint blockade may further lower the required inhibitor dose and widen the therapeutic index [78]. Finally, continued exploration of the non-catalytic functions of AHCY may enable function-selective targeting that circumvents methyl-cycle-associated toxicity [80].

### 7.2. Biomarkers for Disease Diagnosis and Prognosis

SAHH occupies a nodal position linking one-carbon metabolism to methylation homeostasis. Plasma Hcy and the SAH/SAM ratio serve as functional readouts of cellular methylation capacity and are increasingly recognized as causal drivers of disease [88].

#### 7.2.1. Hcy as a Multisystem Risk Indicator

Plasma Hcy is inversely correlated with SAHH activity. Hyperhomocysteinemia is an independent, modifiable risk factor for coronary heart disease, stroke, peripheral vascular disease, and cognitive decline [89,90,91]. The underlying mechanisms are multifactorial and include endothelial dysfunction, vascular smooth-muscle cell proliferation, inflammation and coagulation imbalance [92], together with mitochondrial oxidative stress that contributes to systemic hypertension [93]. In neuropsychiatry, elevated Hcy has been observed in first-episode psychosis irrespective of diagnosis, suggesting a shared early pathological substrate [75]. In ischemic stroke, Hcy interacts with the neuroprotective gasotransmitter H_2_S, and lowering Hcy has been proposed as an adjunctive therapy [94]. Clinically, B-vitamin supplementation effectively reduces Hcy levels [92]. Ultrasensitive detection platforms that couple SAHH activity with electrochemiluminescence have achieved detection limits of 33 fg/mL for carcinoembryonic antigen [95].

#### 7.2.2. SAH and the SAH/SAM Ratio: Direct Indicators of Methylation Potential

SAH and the SAH/SAM ratio more directly reflect methylation capacity. In a prospective cohort of 1553 patients with coronary heart disease, plasma SAH was independently associated with all-cause and cardiovascular mortality (adjusted HR 1.81 and 1.84 for highest vs. lowest quartile, with each SD increase conferring a 25–29% rise in risk) [87]. Mechanistically, SAH accumulation epigenetically up-regulates the long non-coding RNA H19 and inhibits AMPK, drives vascular smooth-muscle cell calcification [96], and induces cellular senescence through NF-κB promoter demethylation [97]. Elevated SAH and reduced SAM/SAH ratios are associated with non-alcoholic fatty liver disease [98], autism spectrum disorder [99], and gestational diabetes mellitus [100]. In the rare inborn error SAH hydrolase deficiency, extreme elevations of SAM and SAH are the diagnostic hallmark; notably, half of the patients present with normal methionine, underscoring the necessity of direct SAH/SAM measurement [101]. A capillary electrophoresis method with laser-induced fluorescence enables one-minute quantification of plasma SAM and SAH [102].

#### 7.2.3. SAHH-Based Biosensing Platforms

The strong feedback inhibition of SAHH by its product adenosine has been used to develop highly selective and sensitive adenosine detection methods with clear diagnostic potential. Ahn et al. [103] designed a fluorescent sensor using DNA-templated copper/silver nanoclusters (DNA-Cu/Ag NCs): Hcy generated by SAHH quenches the nanocluster fluorescence; when adenosine is present, SAHH activity is suppressed, Hcy production halts, and the nanoclusters retain their bright emission. This probe achieves a detection limit as low as 19 nM for adenosine, discriminates efficiently against AMP, ADP, and ATP, and has been applied directly to human serum samples without complicated pretreatment. A second strategy exploits Hg^2+^-mediated thymine–thymine (T-Hg^2+^-T) mismatches to build a molecular beacon: Hcy competitively binds Hg^2+^, disrupting the T-Hg^2+^-T coordination and restoring fluorescence; adenosine, by inhibiting SAHH, reduces Hcy formation and thereby diminishes the signal [104]. This probe exhibits a detection limit of 200 nM, excellent analog discrimination, and applicability to urine and serum samples. These biosensing platforms, which couple the specific catalytic activity of SAHH with nanomaterials or supramolecular recognition, provide novel, cost-effective and pretreatment-free tools for clinical metabolite monitoring, food safety analysis, and environmental contaminant detection, representing an important extension of SAHH applications into analytical biotechnology.

The clinical utility of these biomarkers and biosensing platforms extends beyond diagnosis and risk stratification [87]. Because drug-induced SAH elevation is molecularly indistinguishable from endogenous pathological accumulation, the SAH/SAM ratio could serve as a pharmacodynamic biomarker to provide early safety signals in trials of SAHH-targeted therapies [83,88,105]. Strategies for leveraging this concept to mitigate toxicity are discussed in Section 7.1.

### 7.3. Enzymatic Synthesis of High-Value Compounds

The reversible catalytic activity of SAHH has been exploited to establish green, scalable routes for the synthesis of SAH, adenosine, and their derivatives, offering an attractive alternative to traditional chemical methods [106,107].

#### 7.3.1. SAH and Its Derivatives

The SAHH-catalyzed condensation of adenosine and Hcy to SAH, thermodynamically favored under alkaline conditions, has been developed into a scalable green process for manufacturing SAH and its derivatives. A representative study using the thermostable SAHH from *T. maritima*, overexpressed in *E. coli* and purified by simple heat treatment, achieved a specific activity of 36.8 U/mg at 100 °C and pH 11.2 [60]. Cofactor regeneration, the rate-limiting step, was addressed by coupling the enzyme with an equally thermostable lactate dehydrogenase, boosting SAH yield 6-fold from 24 µmol to 153 µmol at 85 °C. Subsequent innovations include whole-cell biocatalysts co-expressing both enzymes, enzyme immobilization for improved reusability, and continuous-flow processes suitable for pilot-scale production [107]. The enzymatically synthesized SAH is not only a pharmaceutically active compound with sedative and anticonvulsant properties but also serves as the direct precursor for SAM and its analogs, which are essential reagents for methyltransferase research and drug discovery [8,108].

#### 7.3.2. Adenosine and Its Analogs

In the hydrolytic direction, SAHH can be driven to produce adenosine in high yield by the removal of the Hcy co-product or continuous trapping of adenosine under mild aqueous conditions [106,109]. The obtained adenosine is subsequently converted through well-established chemical or enzymatic steps into a panel of approved anticancer nucleoside drugs, including clofarabine, fludarabine phosphate, cladribine and pentostatin, for the treatment of leukemia and certain solid tumors [109,110]. Importantly, these adenosine derivatives are not SAHH inhibitors; their pharmacological activities stem from interference with DNA synthesis, induction of apoptosis, or modulation of adenosine receptors. The SAHH-based adenosine production route thus offers a sustainable, atom-economical alternative to traditional chemical synthesis, and current efforts in protein engineering and process intensification promise to further enhance volumetric productivity and expand substrate scope.

## 8. Concluding Remarks

SAHH is a central regulator of methylation homeostasis, with a catalytic mechanism and domain architecture that are highly conserved across all domains of life. Beyond this conservation, our review highlights the lineage-specific structural adaptations that fine-tune enzyme function: the C-terminal truncation that decouples NAD^+^ affinity from thermostability, the 40-residue insertion that remodels the substrate-binding pocket, and the His-Phe molecular gate whose conformational dynamics are modulated by substrate and metal ions.

The coordinated reduction of the cofactor-binding network in archaeal and thermophilic enzymes, together with the switch in substrate specificity from SAH to SIH, exemplifies an evolutionary trade-off between catalytic efficiency and structural rigidity. These mechanistic insights directly underpin the translational promise of SAHH: species-specific structural features guide selective inhibitor design, the SAH/SAM ratio and Hcy serve as clinical biomarkers, and thermostable variants enable the enzymatic synthesis of high-value pharmaceuticals.

However, it is equally important to recognize that clinical translation remains unproven: no SAHH inhibitor in human trials, biomarker validation incomplete, and industrial bioprocesses still at the laboratory scale. Future research should prioritize three interconnected directions: leveraging lineage-specific pockets for allosteric inhibitor development, integrating SAHH-related metabolites into multi-omics diagnostic panels, and engineering SAHH variants for sustainable biocatalytic production of nucleoside drugs.

## Figures and Tables

**Table 1:** Summary of SAHHs with available structures, including taxonomy, UniProt entry, molecular weight, chain length (single chain), multimeric state, total PDB entries available for the given organism, and representative PDB code. SAHH structures were identified in the RCSB Protein Data Bank using their corresponding UniProt entries.

Taxonomy		UniProt Entry	Mass (KDa)	Chain Length	Multimeric State	Total PDB Entries	Repr. PDB Code	Ref.
Eukaryotes								
	*Cryptosporidium parvum*	Q5CPH1	56	498	homo-4-mer	6	5JPI	[61]
	*Drosophila melanogaster*	Q27580	48	434	homo-4-mer	1	9PDH	[62]
	*Homo sapiens*	P23526	48	435	homo-4-mer	10	3NJ4	[44]
	*Leishmania major*	Q4Q124	48	437	homo-4-mer	1	3G1U	[63]
	*Mus musculus*	P50247	50	452	homo-4-mer	5	8COD	[1]
	*Plasmodium falciparum*	P50250	54	479	homo-4-mer	1	1V8B	[50]
	*Rattus norvegicus*	P10760	47	431	homo-4-mer	7	1B3R	[49]
	*Lupinus luteus*	Q9SP37	54	488	homo-2-mer	3	3OND	[20]
Archaea								
	*Methanococcus maripaludis*	Q6LYR8	48	435	homo-4-mer	1	7R3A	[1]
	*Pyrococcus furiosus*	P50251	50	441	homo-4-mer	5	8QNO	[1]
	*Sulfolobus acidocaldarius*	Q4JAZ7	49	438	homo-4-mer	1	7R39	[1]
Bacteria								
	*Bradyrhizobium elkanii*	A0A087WNH6	53	479	homo-4-mer	6	4LVC	[29,31]
	*Brucella abortus*	Q2YQX8	50	464	homo-4-mer	1	3N58	[64]
	*Burkholderia pseudomallei*	Q3JY79	55	494	homo-4-mer	2	3D64	[65]
	*Cytophaga hutchinsonii*	–	48	438	homo-4-mer	1	6GBN	[66]
	*Elizabethkingia anophelis*	A0A077EDS4	49	445	homo-4-mer	1	6APH	[67]
	*Legionella pneumophila*	Q5ZTY7	48	437	homo-2-mer	5	8WWG	[10]
	*Mycobacterium tuberculosis*	P9WGV3	54	494	homo-4-mer	5	3CE6	[40]
	*Pseudomonas aeruginosa*	Q9I685/ B7V419	51	461	homo-4-mer	50	6F3M	[42]
	*Synechocystis* sp.	P74008	46	425	homo-4-mer/ homo-2-mer	5	7O5M	[36]
	*Thermotoga maritima*	O51933	46	411	homo-4-mer	4	3X2E	[35]

**Table 2:** Clinical development status of representative SAHH inhibitors.

Indication(s)	Preclinical Data	Clinical Status	Key Limitation	Ref.
Neplanocin A				
Broad-spectrum antiviral/anticancer	Potent in vitro and in vivo	Abandoned	Unacceptable systemic toxicity	[85]
DZNep				
Cancer (EZH2 inhibition, B-cell lymphoma)	Broad antitumor activity in mouse models; induces apoptosis via EZH2 inhibition	Stalled/ abandoned	Toxicity; off-target effects	[84]
MSD-914				
Filovirus infection	Full protection in mice; failed in rhesus macaques	Preclinical	Species-dependent efficacy	[74]
DZ2002				
Osteoarthritis, diabetic wounds, dry eye	Rodent efficacy via multiple signaling pathways	Preclinical	No non-human primate or human data	[75,76,77]
Asarinin				
Lung cancer (ferroptosis sensitization)	Reverses ferroptosis resistance in vitro; enhances erastin efficacy in mouse xenograft model	Preclinical	Selectivity and pharmacokinetics unknown	[78]
Compound 7				
Tuberculosis	IC_50_ = 30.2 µM; no Gram-negative toxicity	Preclinical (hit)	Early-stage; no in vivo efficacy	[12]
2-[(3-Chlorobenzoyl)amino]-N-(2,3-dihydro1H-inden-5-yl)-4-methyl-1,3-thiazole-5-carboxamide				
Primary amoebic meningoencephalitis	In silico binding −11.4 kcal⋅mol^−1^; 100 ns MD	Preclinical (in silico)	No experimental validation	[11]
6′-β-fluoro-homoaristeromycin				
Chikungunya virus	EC_50_ = 0.12 µM	Preclinical	Limited in vivo data	[79]

## Data Availability

No new data were created or analyzed in this study. Data sharing is not applicable to this article.

## Supplementary Materials

The following supporting information can be downloaded at: https://www.mdpi.com/article/10.3390/biom16071010/s1, Table S1. Systematic functional interpretation of conserved interactions of SAHH from plant (*Lupinus luteus*, PDB code 3OND [20]), bacteria (*Pseudomonas aeruginosa*, PDB code 6F3M [42]), animal (*Homo sapiens*, PDB code 3NJ4 [44]), and archaeon (*Sulfolobus acidocaldarius*, PDB code 7R39 [1]) with substrate (adenosine) and cofactor (NAD^+^). Asterisks indicate residues originating from the neighboring subunit. Figure S1: Multiple sequence alignment of SAHH from representative species across archaea, eukaryotes, and bacteria.

## Abbreviations

The following abbreviations are used in this manuscript:

SAHH	*S*-adenosyl-L-homocysteine hydrolase
SAH	*S*-adenosyl-L-homocysteine
SAM	*S*-adenosylmethionine
SIH	*S*-inosyl-L-homocysteine
Hcy	Homocysteine
DTNB	5,5′-dithiobis-(2-nitrobenzoic acid)
TNB^2−^	2-nitro-5-thiobenzoate
HPLC	High-performance liquid chromatography
*Tm*SAHH	Thermotoga maritima SAHH
*Hs*SAHH	*Homo sapiens* SAHH
*Ll*SAHH	*Lupinus luteus* SAHH
*Lp*SAHH	*Legionella pneumophila* SAHH
*Be*SAHH	*Bradyrhizobium elkanii* SAHH
*Pa*SAHH	*Pseudomonas aeruginosa* SAHH
*Sa*SAHH	*Sulfolobus acidocaldarius* SAHH
DZNep	3-deazaneplanocin A
