# Supplementary material for: S-Adenosyl-L-Homocysteine Hydrolase (SAHH): Structure, Function, and Applications
Source: Biomolecules. 2026 Jul 10;16(7):1010. doi: 10.3390/biom16071010 (PMC13406329; doi:10.3390/biom16071010)
Supplement: Supplementary file 1 [file biomolecules-16-01010-s001.zip › biomolecules-4308813-supplementary.pdf]

Supplementary Materials

S-Adenosyl-L-Homocysteine Hydrolase (SAHH): Structure, Function, and Applications

Jinsha Huang[1,2], Qingpu Chen[2], Haihua He[2], Kai Du[2,*], Zhangli Hu[1,*]

1 College of Life Sciences and Oceanography, Shenzhen University, Shenzhen 518060, China;
2 Research & Development Department, Shenzhen New Industries Biomedical Engineering Co., Ltd. (Snibe), Shenzhen 518122, China;
* Correspondence: Kai Du, kai.du@snibe.cn; Zhangli Hu, huzl@szu.edu.cn;

Table of Contents

Table S1. Systematic functional interpretation of conserved interactions of SAHH from plant (*Lupinus luteus*, PDB code 3OND [20]), bacteria (*Pseudomonas aeruginosa*, PDB code 6F3M [42]), animal (*Homo sapiens*, PDB code 3NJ4 [44]), and archaeon (*Sulfolobus acidocaldarius*, PDB code 7R39 [1]) with substrate (adenosine) and cofactor (NAD^+). Asterisks indicate residues originating from the neighboring subunit.

Figure S1: Multiple sequence alignment of SAHH from representative species across archaea, eukaryotes, and bacteria.

Table S1. Systematic functional interpretation of conserved interactions of SAHH from plant (*Lupinus luteus*, PDB code 3OND [20]), bacteria (*Pseudomonas aeruginosa*, PDB code 6F3M [42]), animal (*Homo sapiens*, PDB code 3NJ4 [44]), and archaeon (*Sulfolobus acidocaldarius*, PDB code 7R39 [1]) with substrate (adenosine) and cofactor (NAD^+). Asterisks indicate residues originating from the neighboring subunit.

Interaction site	Key conserved residue					Interaction type	Function
	L. luteus	*P. aeruginosa*	*H. sapiens*	*S. acidocaldarius*	Atom/group		
☞**Substrate**							
**Ribofuranose**							
5'-OH	D139	D139	D131	D124	Oδ1	H-bond	Stabilizes ribose prior to oxidation/catalysis
5'-OH	H62	H61	H55	H51	Nε2	H-bond	
3'-OH	T206	T165	T157	T148	Oγ1	H-bond	Stabilize ribose ring positioning
3'-OH	K235	K194	K186	K177	Nζ	H-bond	
2'-OH	E205	E164	E156	E147	Oε2	H-bond	Constrain ribose in C4′-endo conformation for hydride transfer
2'-OH	D239*	D198*	D190*	D181*	Oδ2	H-bond	
**Adenine ring**							
heterocyclic N1	T64	T63	T57	T53	Oγ1	H-bond	Maintains purine ring planarity
exo-N6	Q66	Q65	E59	Q55	Sidechain O	bidentate H-bond	Locks adenine in anti-conformation
exo-N6	H404	H382	H353	H344	main-chain O	H-bond	
heterocyclic N7					main-chain N		Additional ring stabilization
Adenine ring	M409	M387	M358	M349	sidechain	C–H···π	Clamps adenine base and enhances binding affinity
	L398	L376	L347	L338		hydrophobic interaction	

☞**Cofactor**

Interaction site	Key conserved residue					Interaction type	Function
	L. luteus	*P. aeruginosa*	*H. sapiens*	*S. acidocaldarius*	Atom/group		
**Adenine**							
Puring ring	I293	V252	I244	S235	backbone	C-H···π	Anchors adenine in anti-conformation
	T325	T298	T276	T267	backbone	hydrophobic interaction	
N7	N327	N300	C278	N269	Nδ2	H-bond	
**Nicotinamide**							
N7	I348	I321	I299	S290	main-chain O	H-bond	Locks nicotinamide orientation for hydride transfer
N7	N397	N375	N346	N337	Oδ1	H-bond	
O7					Nδ2	H-bond	
**Adenosine ribofuranose**							
2'-OH, 3'-OH	E292	E251	E243	E234	Oε	bidentate H-bonds	Primary ribose lock;
	K479*	K463*	K426*	/	Nζ	bidentate H-bonds	
**Nicotinamide ribofuranose**							
2′-OH	T206	T165	T157	T148	Oγ1	H-bond	Locks ribose in C2′-endo; Positions C4 for catalysis
2′-OH, 3′-OH	T208	T167	T159	T150	Oγ1	bidentate H-bonds	
**Phosphate groups**							
Oα1	D272	D231	D223	W214	main-chain N	electrostatic interaction	Stabilizes phosphates;
Oβ	T207	T166	T158	T149	Oγ1	polar interaction	
Oβ	N240	N199	N191(Oδ2)	N182	Nδ2	H-bond	
Oα1	Y483*	Y467*	Y429*	/	phenol-OH	polar interaction	

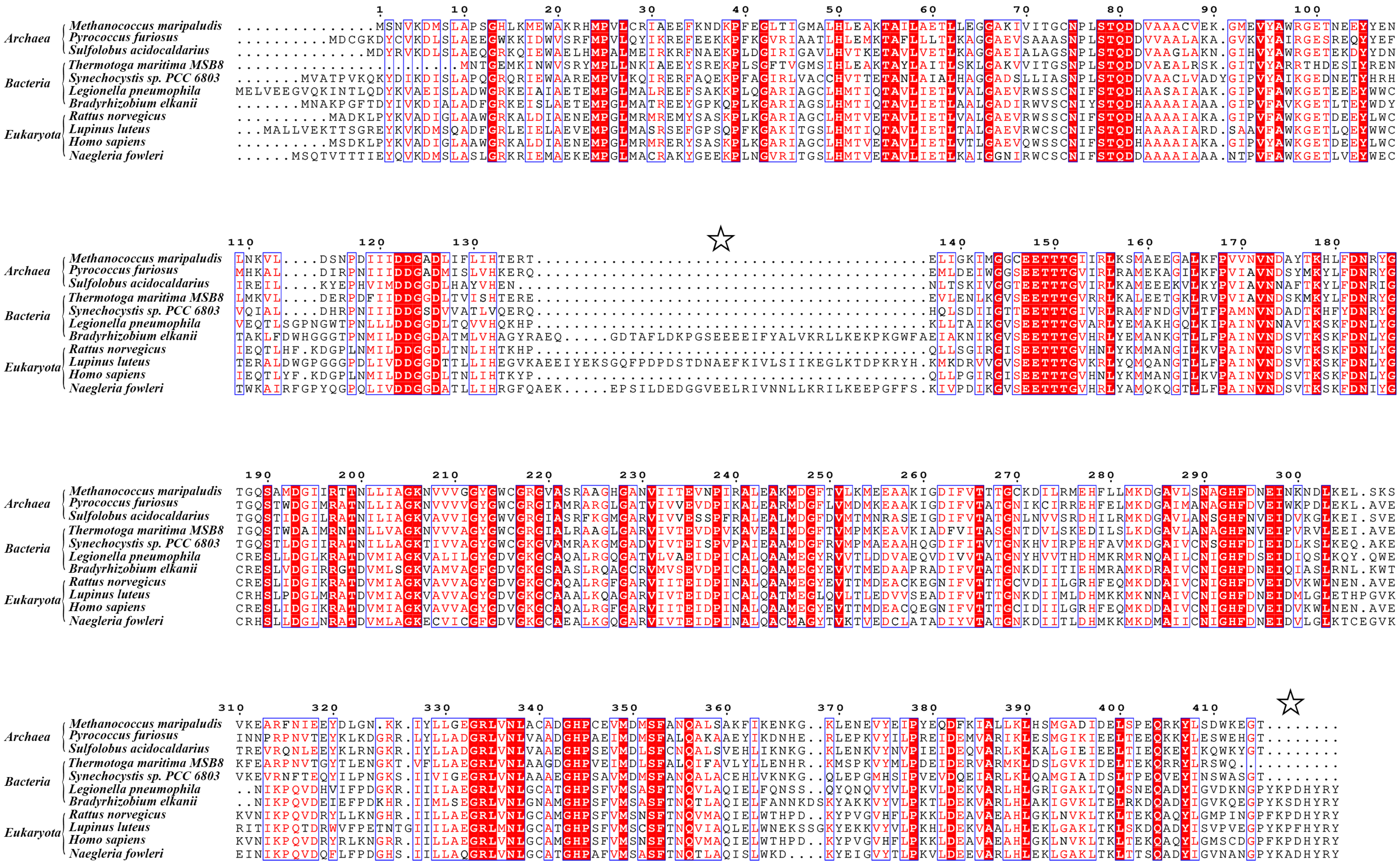

Figure S1: Multiple sequence alignment of SAHH from representative species across archaea, eukaryotes, and bacteria.
